# Preparation of Salvianolic Acid B Disodium Salt Considering the Water Extract Quality Standard

**DOI:** 10.3390/molecules24071269

**Published:** 2019-04-01

**Authors:** Tiantian Ye, Haibin Qu, Xingchu Gong

**Affiliations:** Pharmaceutical Informatics Institute, College of Pharmaceutical Sciences, Zhejiang University, Hangzhou 310058, China; 11319023@zju.edu.cn (T.Y.); quhb@zju.edu.cn (H.Q.)

**Keywords:** crude SAB preparation, quality standard of water extract, salvianolic acid B disodium salt, process optimization

## Abstract

A preparation process of salvianolic acid B (SAB) disodium salt from *Salvia miltiorrhiza Bunge* (Danshen) is provided in this work. A water extract quality standard was also developed to estimate the influences of Danshen quality on SAB disodium salt quality at an early stage of the preparation process. Crude SAB solution was obtained after water extraction, concentration, acidification, 1-butanol extraction, water washing, basification, and water back extraction. Extraction temperature, extraction pH, and back-extraction pH were identified to be key parameters for the preparation of crude SAB solution. These parameters were optimized with Box–Behnken designed experiments. Crude SAB solution was further purified with a chromatography process. AMBERCHROW CG161M resin was selected as the best adsorbent. SAB disodium salt could be obtained by drying the eluate. Considering the quality of Danshen may affect the purity and yield of SAB disodium salt, different batches of Danshen were used to prepare SAB disodium salt with the optimized parameters. Water extract indices of phenolic compound purity and phenolic compound yield were measured. By developing models between SAB disodium salt purity and yield with water extract indices, the quality standard of Danshen water extract was obtained. The application of water extract quality standards can improve the quality consistency of SAB disodium salt. The effects of different batches of Danshen raw materials on the final product could be evaluated at the beginning of production stages. The present method could prepare about five grams of high-purity SAB disodium salt (>95%) in one preparation cycle. The method reported in this work can also be used to develop process intermediate quality standards for other natural products.

## 1. Introduction

Danshen, the dried root of *Salvia miltiorrhiza Bunge*, is one of the most popular Chinese medicinal plants [1]. Phenolic acids and tanshinones are major active components in Danshen [2,3]. Salvianolic acid B (SAB) is the most abundant among the phenolic acids [4] with the bioactivities as an antioxidant [5,6], improving regional cerebral blood flow [7], protection against atherosclerosis [8], and so forth. 

There are many publications on the purification of SAB from Danshen, with different technologies of liquid–liquid extraction [9], high-speed countercurrent chromatography [10,11,12,13], and resin chromatography [14,15,16]. Nie et al. [17] used ionic liquid-modified silica gel for the separation of water-soluble phenolic acids, including SAB. Guo et al. [18] reported a two-step procedure for the large-scale purification of SAB using an aqueous two-phase extraction followed by preparative high-performance liquid chromatography (HPLC). Li et al. [19] developed a method of online hyphenation of expanded bed adsorption chromatography and countercurrent chromatography for extraction and purification of SAB from Danshen. 

The publications on SAB preparation focused on the development of preparation processes. However, the effect of Danshen quality was not considered. Zhong et al. [20] found that medicinal herb quality may show a greater impact on herbal product quality than that of the production process. The analysis of the chemical marker contents of a small amount of medicinal herbs is easy. However, medicinal herbs are heterogeneous. There may be a remarkable difference between the chemical marker contents obtained from a small sample analysis and the overall average of a large number of herbs. It indicates that accurate measurement of herb quality is not easy. An alternative method is to determine the chemical marker contents in the solvent extract of medicinal herbs, which is usually a homogeneous solution. 

**Figure 1:**
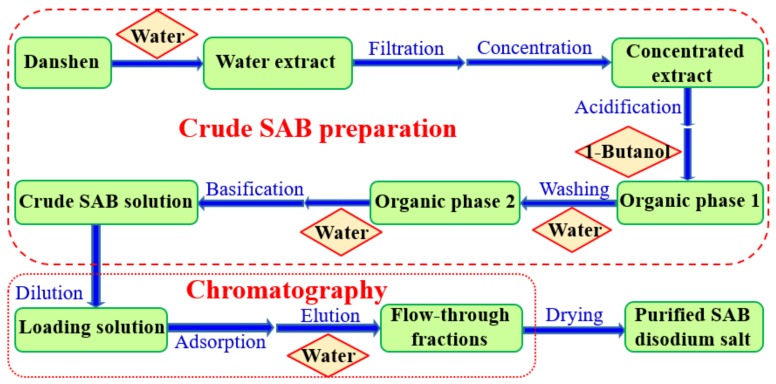
Schematic diagram of the preparation of SAB disodium salt.

In this work, the preparation process of SAB disodium salt was developed and is shown in Figure 1. Crude SAB solution was prepared via water extraction, concentration, acidification, 1-butanol extraction, water washing, basification, and water back-extraction. After that, SAB disodium salt was obtained using a chromatography process and drying. Water extract was considered as a critical intermediate because its features reflect the impacts of Danshen materials and the water extraction process. If the water extract is not up to standard, there will be no need to carry out downstream purification processes. 

**Figure 2:**
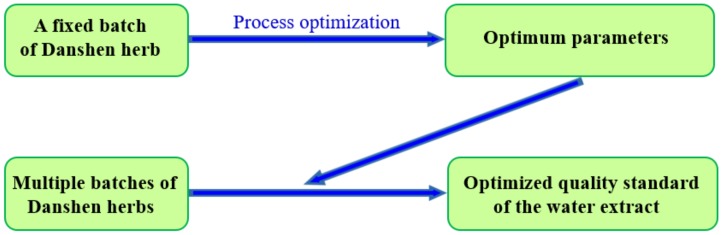
Flow chart of the development of the quality standard for a water extract.

In order to develop water extract standards, two procedures were carried out, as shown in Figure 2. First, process parameters were optimized with the same batch of Danshen herb. Second, different batches of Danshen herbs were processed with the optimized process parameters. The features of water extracts and SAB disodium salt products were determined. After modeling and calculation, the standard of water extracts was obtained. A verification experiment was carried out. 

## 2. Results and Discussion

### 2.1. Crude SAB Preparation

The Plackett–Burman designed experiments and results are presented in Table 1. The results of partial regression coefficients, importance factors, and coefficient confidence intervals are listed in Table S1. The parameters with the importance factor (*IF_j_*, subscript *j* refers to a process parameter) value ranked in the top three of all the seven parameters of crude SAB preparation process were selected as key parameters. Therefore, water extraction temperature, 1-butanol extraction pH, and back-extraction pH were selected as key parameters of the crude SAB preparation process.

Table 2 displays the results of Box–Behnken designed experiments. SAB yield in crude SAB solution(${SAB}_{yield}^{S}$, superscript S refers to the crude SAB solution) was between 0.227 and 10.5 mg/g medicinal slices. SAB purity in crude SAB solution (${SAB}_{purity}^{S}$) varied from 43.5% to 59.9%. The estimated parameter values, determination coefficients (R^2^), ANOVA results, and coefficient confidence intervals of quadratic models are shown in Table S2. The values of R^2^ of the two models were higher than 0.85. The two models were significant with *p*-values less than 0.01. ${SAB}_{purity}^{S}$ increased as extraction temperature decreased.

**Figure 3:**
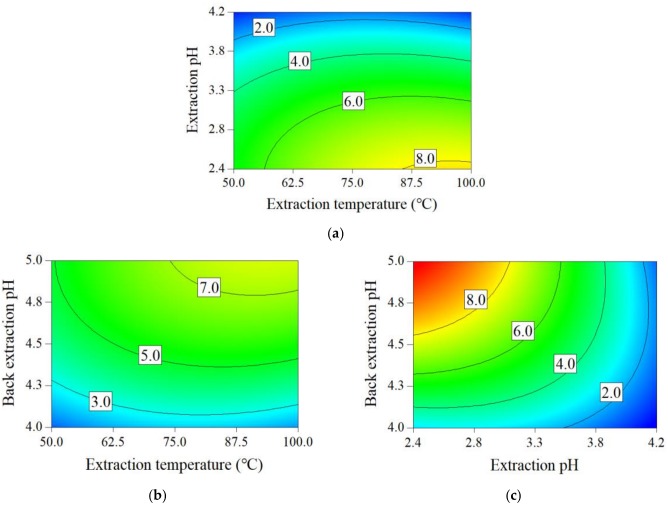
Contour plots for key process parameter effects on the ${SAB}_{yield.}^{S}$ (**a**) Extraction pH and extraction temperature effect on the ${SAB}_{yield}^{S}$ when back-extraction pH = 4.5, (**b**) back-extraction pH and extraction temperature effect on the ${SAB}_{yield}^{S}$ when extraction pH = 3.3, (**c**) back-extraction pH and extraction pH effect on the ${SAB}_{yield}^{S}$ when extraction temperature = 75.0 °C.

**Figure 4:**
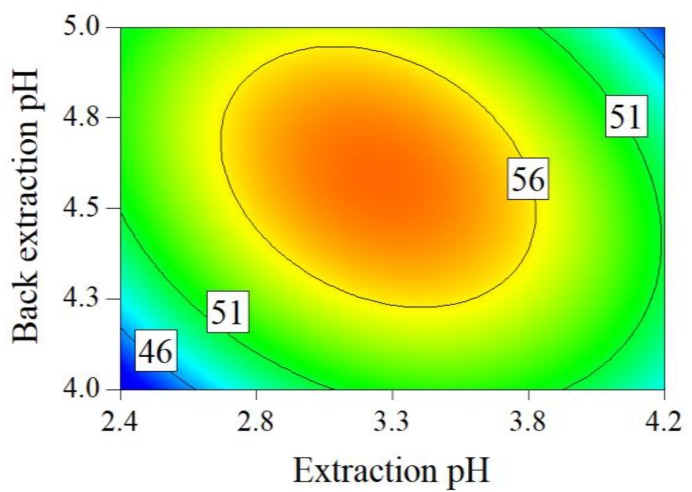
Contour plots for key process parameter effects on the ${SAB}_{purity}^{S}$ (extraction temperature = 75.0 °C).

The contour plots of the obtained mathematical models for the ${SAB}_{yield}^{S}$ and ${SAB}_{purity}^{S}$ are displayed in Figure 3 and Figure 4. In Figure 3a–c, higher extraction temperature, higher back-extraction pH, and lower extraction pH led to a higher ${SAB}_{yield}^{S}$, respectively. 

The optimized parameter ranges for the crude SAB preparation process are as follows: the extraction temperature was from 60 °C to 80 °C, the extraction pH was from 2.5 to 3.0, and the back-extraction pH was from 4.6 to 5.0. The ${SAB}_{purity}^{S}$ and ${SAB}_{yield}^{S}$ were more than 50.0% and higher than 5.0 mg/g medicinal slices under the above optimized parameters.

### 2.2. SAB Purification with Chromatography Process

#### 2.2.1. Resin Screening by Adsorption Capacity, Desorption Capacity, and Desorption Ratio

**Figure 5:**
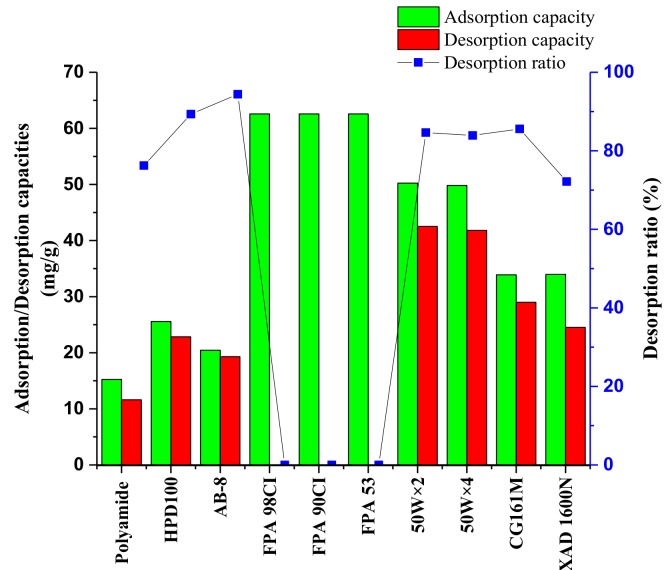
Adsorption and desorption capacities, and the desorption ratio of SAB on ten resins.

The adsorption and desorption capacities of different resins for the SAB were shown in Figure 5. High adsorption capacity (>60 mg/g dry resin) were observed for the resins of FPA98Cl, FPA90Cl, and FPA53. However, the adsorbed SAB could hardly be desorbed with ethanol solution. The results were reasonable because these three resins are anion exchange resins. The adsorption capacities of polyamide, HPD100, and AB-8 resins were lower than 30 mg/g dry resin. The adsorption capacity of 50 W × 2, 50 W × 4, CG161M, and XAD 1600N were higher than 30mg/g dry resin. The desorption ratio of 50 W × 2, 50 W × 4, and CG161M were higher than 80%. Therefore, 50 W × 2, 50 W × 4, and CG161M were chosen to be further investigated.

#### 2.2.2. Resin Screening by Adsorption Kinetics 

**Figure 6:**
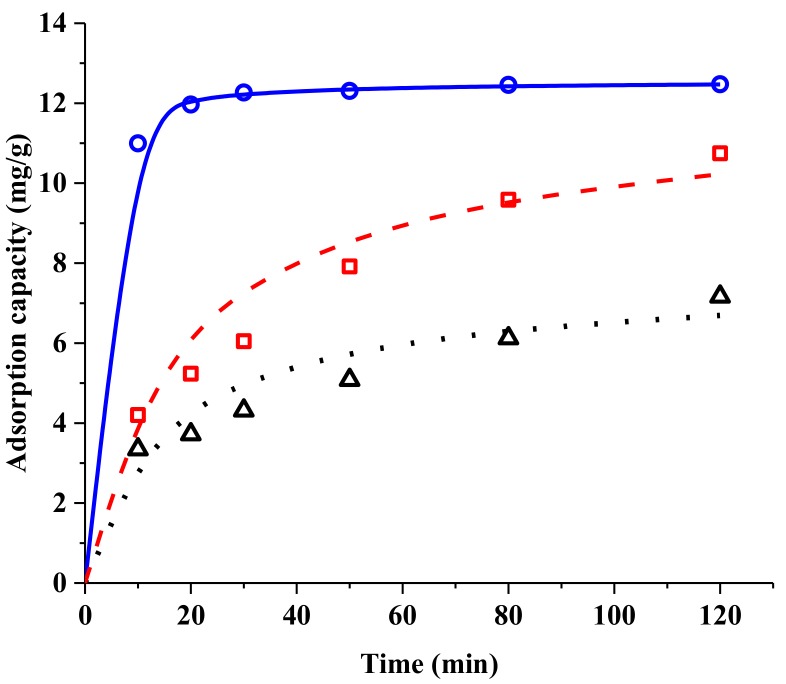
Adsorption kinetics of SAB fitted to pseudo-second-order model. Solid line, CG161M resin, (calc.); dashed line, 50 W × 4 resin, (calc.); dotted line, 50 W × 2 resin, (calc.); **○**, CG161M resin, (exp.); **□**, 50 W × 4 resin, (exp.); **△**, 50 W × 2 resin, (exp.).

Adsorption kinetics of SAB were measured with CG161M, 50 W × 2, and 50 W × 4 resins at 30 °C. The results are shown in Figure 6. The adsorption amount of SAB increased with the adsorption time for all three resins. For resin CG161M, equilibrium was almost reached after 30 min. For the other two resins, at least 120 min was required to reach equilibrium. 

The pseudo-second-order model was used to fit the experimental results. In Table 3, all the R^2^ values are higher than 0.95. The values of *k*_2_ for CG161M, 50 W × 2, and 50 W × 4 resins were 0.103 min^−1^, 0.00900 min^−1^, and 0.00479 min^−1^, respectively. The highest *k*_2_ value for CG161M meant it had the fastest absorption rate among the three resins. Therefore, the CG161M resin was selected for the purification of SAB. 

#### 2.2.3. Chromatographic Process 

**Figure 7:**
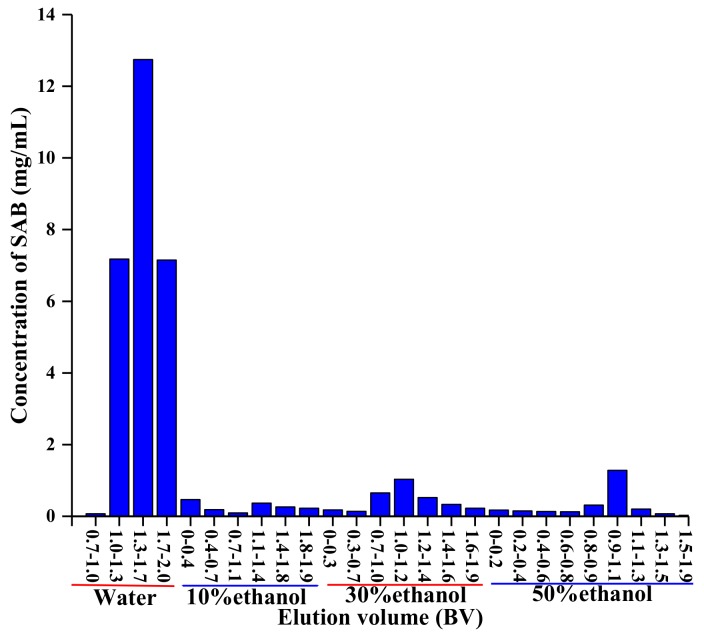
Elution profile of SAB on CG161M resin.

After loading the diluted crude SAB solution to the CG161M column, the column was eluted with 2 bed volume (BV) of deionized water, 2 BV of 10% *v*/*v* ethanol, 2 BV of 30% *v*/*v* ethanol, and 2 BV of 50% *v*/*v* ethanol sequentially. The flow-through elution fractions were collected. The concentrations of SAB in the elution fractions were analyzed and are shown in Figure 7.

More than 78.0% SAB was collected from 1.0–2.0 BV water elution fractions. In contrast, only 14.6% SAB was collected from 10% to 50% ethanol (*v*/*v*) elution fractions. Most of the SAB was collected within water elution fractions. Thus, water was chosen as the desorption solvent for the purification of SAB. Elution volume of 1.0–2.0 BV was collected. The resin was regenerated with 90% ethanol (*v*/*v*) for 8 BV and water for 8 BV, respectively.

### 2.3. Water Extract Standard

The yield and purity of different phenolic compounds, including Danshensu (DSS), protocatechuic aldehyde (PA), caffeic acid (CA), rosmarinic acid (RA), lithospermic acid (LA), SAB, and salvianolic acid A (SAA), were used as quality indices of Danshen water extracts. The data are shown in Table 4. The corresponding values of the yield of SAB in final product (${SAB}_{yield}^{F}$) and the purity of SAB in final product (${SAB}_{purity}^{F}$) were also listed in Table 4. Stepwise regression was used to build the models between ${SAB}_{yield}^{F}$ and ${SAB}_{purity}^{F}$ data and water extract indices of phenolic compound purity (${PC}_{purity}^{E}$) and phenolic compound yield (${PC}_{yield}^{E}$). The *p*-values for adding and removing a term were set to 0.01. The Design Expert 10.0.4.0 software (Stat-Ease Inc., Minneapolis, MN, USA) was used for the calculations. ${SAB}_{yield}^{F}$ or ${SAB}_{purity}^{F}$ was assumed to be only affected by the phenolic compound yield or purity in water extracts. The obtained models were as follows:

(1) $$SAB_{purity}^{F}=1.079-543.7\times CA_{purity}^{E}\;R^{2}=0.9546;$$

(2) $$SAB_{yield}^{F}=0.04756\times SAB_{yield}^{E}-0.002992\;R^{2}=0.7668$$

where superscripts *E* and *F* refer to the water extract and the final product, respectively. The determination coefficients of regression Equations (1) and (2) were all higher than 0.76, which implied that most variations of experimental data can be explained. 

In this work, a prediction error propagation method was used to estimate the water extract standards [21]. Taking Equation (1) as an example, the residuals were assumed to follow a normal distribution with a mean value of zero. The standard deviation of this normal distribution for the Equation (1) ($SD_{Residual}^{Eq1}$) was assumed to be the same of that of residuals, which was 0.05575. If ${SAB}_{purity}^{F}$ was expected to be not lower than 90% with a probability of 80%, the following equation was used to calculate maximum ${CA}_{purity}^{E}$ value.

(3) $$SAB_{purity}^{F}=1.079-543.7\times CA_{purity}^{E}-Z_{0.2}\times SD_{Residual}^{Eq1}\geq 0.900$$

where $Z_{0.20}$ was the normal distribution boundary, which equaled 0.84. Then, ${CA}_{purity}^{E}$ should be lower than 2.43 × 10^−4^.

By using the same method, the standard deviation of the normal distribution for Equation (2) ($SD_{Residual}^{Eq2}$) was assumed to be equivalent to that of the residuals, which was 0.1894. If ${SAB}_{yield}^{F}$ was expected to be not lower than 0.300 mg/g medicinal slices with a probability of 80%, the following equation was used to calculate the minimum ${SAB}_{yield}^{E}$ value:

(4) $$SAB_{yield}^{F}=0.04756\times SAB_{yield}^{E}-0.002992-Z_{0.2}\times SD_{Residual}^{Eq2}\geq 0.300$$

where $Z_{0.20}$ was the normal distribution boundary, which equaled 0.84. Then, ${SAB}_{yield}^{E}$ should be higher than 9.72 mg/g medicinal slices.

### 2.4. Verification

Verification experiments are displayed in Table 5. The conditions of the verification experiments were the same as the mentioned conditions in Section 3.4. The experimental value of ${SAB}_{purity}^{F}$ was 99.3% and agreed well with the prediction value. The experimental value of ${SAB}_{yield}^{F}$ was higher than 0.700 mg/g medicinal slices. These results indicated that the quality standards of Danshen water extract were reliable. 

### 2.5. Identification of SAB Disodium Salt

After drying, a tan amorphous powder was obtained. The Fourier transform infrared spectroscopy (FTIR) measurements were carried out with a Fourier transform infrared spectrophotometer (JASCO, FT-IR4100, Tokyo, Japan) in the wavenumber range of 500–4000 cm^−1^. With the FTIR spectrum in Figure S1, the peak at 3423 cm^−1^ was assigned to the hydroxyl stretching vibration [22]. Compared to the carbonyl peaks of SAB (1726 cm^−1^, 1613 cm^−1^, 1521 cm^−1^, and 1445 cm^−1^), the four carbonyl peaks of this compound (1719 cm^−1^, 1590 cm^−1^, 1520 cm^−1^, and 1440 cm^−1^) with the low wavenumber shift may have been caused by the formation of the carboxylate. The absorption peaks of 1262 cm^−1^ and 1167 cm^−1^ were attributed to the characteristic vibration of the hydroxyl group on the benzene ring [22].

A Triple TOF 5600+ mass spectrometer (AB SCIEX, Framingham, MA, USA) provided useful information for the assessment of the cation, which is shown in Figure S2. The time-of-flight mass spectrometer (TOF-MS) fragments at m/z 761 of this compound suggested the presence of [SAB − H]^–^ (*m*/*z* 717) and two sodium ions. Hence, it was deduced that this powder might be a disodium salt of SAB.

An aqueous solution of the obtained powder was prepared at a concentration of 9.832 mg/L. The Na^+^ concentration of aqueous solution was then determined using an atomic absorption spectrophotometer (240AA, Agilent, PA, CA, USA). The concentration of sodium ions was 0.5955 mg/L. If this powder was SAB disodium salt, the theoretical sodium ion concentration should be 0.5928 mg/L, which is very close to the measured value.

Consequently, this powder was determined to be SAB disodium salt.

## 3. Materials and Methods

### 3.1. Materials and Reagents

Danshen *(Salvia Miltiorrhiza)* were originally collected from six different habitats in China (Bozhou, Anhui Province; Wanrong, Shanxi Province; Minxian, Gansu Province; Dancheng, Henan Province; Zhongjiang, Sichuan Province, and Julu, Hebei Province). Seven reference compounds, including sodium Danshensu (DSS-Na, lot. 180327, 98.92%), PA (lot. 171126, 99.69%), CA (lot. 180105, 99.62%), RA (lot. 180403, 99.47%), LA (lot. 180107, 98.85%), SAB (lot. 180120, 99.36%), and SAA (lot. 171229, 98.94%) were purchased from Shanghai Winherb Medical Science (Shanghai, China) as standard compounds without further purification. HPLC-grade formic acid was obtained from ROE Scientific Inc. (Newark, DE, USA). HPLC-grade methanol and acetonitrile were purchased from Merck (Darmstadt, Germany). Analytical grade 1-butanol was supplied by Sinopharm Chemical Reagent (Beijing, China). Analytical grade ethanol was supplied by Shanghai Lingfeng Chemical Reagent (Shanghai, China). Analytical grade hydrochloric acid and sodium hydroxide were purchased from Sinopharm Chemical Reagent (Beijing, China). Deionized water was prepared through a water purification system (Milli-Q, Milford, MA, USA). Macroporous resins of HPD100 and AB-8 were purchased from CangZhou Bonchem Co., Ltd. (Hebei, China) and Zhengzhou Qinshi Technology (Guangdong, China), respectively. Polyamide resin (30–60 mesh) was supplied by Taizhou Luqiao Sijia Biochemical Plastics Factory (Zhejiang, China). The resins including AMBERLITE FPA98Cl, AMBERLITE FPA90Cl, AMBERLITE FPA53, AMBERLITE XAD 1600N, DOWEX 50 W × 2, DOWEX 50 W × 4, and AMBERCHROW CG161M were obtained from Dow Chemical Company (Midland, MI, USA). 

### 3.2. Optimization of Crude SAB Preparation 

#### 3.2.1. Procedures

Deionized water was added to 150 g of the Danshen under mechanical stirring. The water extract was then filtered and concentrated under reduced pressure. After a small volume of 5 M HCl solution was added, the liquid–liquid extraction was carried out by contacting the concentrated extract of Danshen with 1-butanol. The pH values were monitored by a pH meter (S40 SevenMulti, Mettler-Toledo GmbH, Greifensee, Switzerland) equipped with a combination pH electrode (InLab Expert Pro, Mettler-Toledo GmbH, Greifensee, Switzerland). After magnetic stirring (85-1, Hangzhou Instrument Motor Co., Ltd., Hangzhou, Zhejiang, China) for 0.5 h, the mixture was centrifuged for 10 min at a rotation speed of 3000 rpm using a centrifuge (5810R, Eppendorf, Westbury, NY, USA) to separate the two phases. The upper phase was collected and named as Organic Phase 1. Organic Phase 1 was then contacted with deionized water in a separatory funnel. After vigorously shaking, the mixture then was centrifuged for 10 min at a rotation speed of 3000 rpm to separate two phases. The upper phase was collected again and named as Organic Phase 2. After Organic Phase 2 was collected into a conical flask, deionized water and a small volume of 4% NaOH (*w*/*w*) solution were added. The mixture was then magnetically stirred for 0.5 h, and then centrifuged. Finally, the aqueous phase was collected and considered to be the crude SAB solution. The schematic diagram of crude SAB preparation is presented in Figure 1.

The crude SAB solution was further purified using a chromatography process. Adsorbents were selected. The elution process was also optimized. The flow-through fractions were concentrated under reduced pressure at 30 °C. Finally, SAB disodium salt was obtained by freeze-drying (MICROMODULYO230, Thermo Electron Corporation, Walthan, MA, USA). The schematic diagram of chromatographic processes and drying were also shown in Figure 1.

#### 3.2.2. Experimental Design

To screen significant variables in the preparation of crude SAB, Plackett–Burman-designed experiments were carried out with the conditions displayed in Table 1. Seven parameters were investigated, including extraction temperature, extraction pH, back-extraction pH, extraction time, water consumption for extraction, 1-butanol amount, and water consumption for washing. Box–Behnken designed experiments were used to investigate the relationships between process indices and key parameters of crude SAB preparation process, as shown in Table 2.

### 3.3. Optimization of Chromatography

#### 3.3.1. Selection of Resins

##### Adsorbents

Resins were weighed, then put into a drying oven, and dried at 60 °C to a constant weight. After that, their moisture was calculated. The physical properties of the resins are given in Table S3. The resins of polyamide resin, HPD100, AB-8, CG161M, and XAD 1600N were washed with ethanol and deionized water sequentially before used. The resins, including FPA98Cl, FPA90Cl, 50 W × 2, and 50 W × 4, were pretreated using 1 mol/L HCl, 1 mol/L NaOH, 1 mol/L HCl, and water. FPA53 were pretreated using 1 mol/L HCl, 1 mol/L NaOH, and water in a sequential manner.

##### Static Adsorption and Desorption Tests 

The crude SAB solution was diluted 10 times (*w*/*w*) with deionized water. Resins of 1 g dry weight and 25 mL diluted crude SAB solution were added into a 50 mL conical flask. The flasks were placed in a temperature-controlled oscillator (DSHZ-300, Taicang City Experimental Equipment Factory, Jiangsu, China), shaken at 140 rpm at 30 °C for 20 h. After adsorption, the resins were desorbed with 40 mL 70% ethanol (*v*/*v*) solution and shaken at 140 rpm at 30 °C for 10 h. The concentration of SAB in the solutions after adsorption and desorption were sampled and analyzed using HPLC, respectively.

To select the proper resin, the static adsorption/desorption capacity and ratio of desorption were calculated using the Equations (5)–(7) below:

(5) $$Q_{e}=\frac{\left(C_{i}-C_{e}\right)V_{i}}{W}$$

(6) $$Q_{d}=\frac{C_{d}V_{d}}{W}$$

(7) $$R_{d}=\frac{C_{d}V_{d}}{\left(C_{i}-C_{e}\right)V_{i}}\times 100\%$$

where *Q_e_* (mg/g dry resin) represents the adsorption capacity under equilibrium; *Q_d_* (mg/g dry resin) represents the desorption capacity; *R_d_* (%) represents the desorption ratio; *C_i_* and *C_e_* (mg/mL) represent the initial and equilibrium concentration of SAB in the solution, respectively; *V_i_* (mL) represents the volume of the initial solution; *W* (g) represents the dry weight of the tested resins; *C_d_* (mg/mL) represents the concentration of SAB in the desorption solution; and *V_d_* (mL) represents the volume of the desorption solution.

#### 3.3.2. Adsorption Kinetics Tests

The crude SAB solution was diluted 50 times (*w*/*w*) with deionized water. After that, resins of 1 g dry weight and 35 mL dilution were added into a 50 mL conical flask. The flasks were placed in a temperature-controlled oscillator and shaken at 140 rpm at 30 °C for 2 h. A total of 0.2 mL of solution was sampled at 10, 20, 30, 50, 80, and 120 min to measure the SAB concentration. In order to evaluate the effect of contact time on the adsorption kinetics, the pseudo-second-order model [23,24,25] was used as followed:

(8) $$\frac{t}{Q_{t}}=\frac{1}{k_{2}Q_{e}^{2}}+\frac{t}{Q_{e}}$$

where *Q_t_* (mg/g dry resin) is the amount of SAB adsorbed per gram of dry resin at time *t*, and *k*_2_ is the rate constant of pseudo-second-order kinetics model.

#### 3.3.3. Optimization of the Chromatographic Process

Crude SAB solution was diluted five times (*w*/*w*) with deionized water. Adsorption of SAB in the diluted crude SAB solution was carried out in a glass column (450 mm × 60 mm) packed with the optimized resin. After being washed with 90% *v*/*v* ethanol solution and equilibrated with deionized water, the column was loaded with diluted crude SAB solution at a constant flow rate of 1.94 BV/h. The column was then desorbed with 2 BV deionized water, 10% *v*/*v* ethanol, 30% *v*/*v* ethanol, and 50% *v*/*v* ethanol sequentially at a constant flow rate of 1.94 BV/h. The flow-through fractions were collected at intervals. The SAB concentrations and dry matter contents of the samples were determined. The eluting solvent of chromatographic process and the collection conditions were optimized according to experimental results.

### 3.4. Standard Establishment of the Water Extract

The medicinal slices of Danshen from five different producing areas were processed to obtain SAB disodium salt. A total of 450.0 g medicinal slices of Danshen were extracted in 3600 mL of water under mechanical stirring at 69 °C for 2.0 h. The water extraction process was repeated once more under the same conditions. The extracts were combined, filtered, and then concentrated at 63 °C to 1800 mL. The concentrated extract was acidified using HCl solution to a pH value of 2.70. After that, the concentrated extract was contacted with 1-butanol. Then Organic Phase 1 was collected. After washing with water, Organic Phase 2 was collected. Organic Phase 2 was mixed with deionized water, and then basified using a NaOH solution to a pH value of 5.00. The lower phase was collected as a crude SAB solution. Crude SAB solution was diluted five times (*w*/*w*) with deionized water. The diluted crude SAB solution was then loaded to the resin column at a constant flow rate of 1.0 BV/h, and then desorbed with deionized water. The flow-through fractions were collected at intervals. After that, the collected flow-through fractions were dried, and finally, the SAB disodium salt was obtained. Seven phenolic compound yield values and purity values were measured to represent water extract features. The SAB yield value and purity value in the final product were also determined. The relationships between water extract features and SAB disodium salt indices were analyzed with the stepwise regression. After that, the quality standards of the water extract were obtained. A new batch of Danshen was extracted and purified to verify the water extract quality standards. 

### 3.5. Analytical Methods

The quantitative measurement of DSS, PA, CA, RA, LA, SAB, and SAA were carried out via HPLC analysis according to the method published by Cao et al. [4]. Two HPLC systems were used, including HP 1100 series (Agilent Technologies, Palo Alto, CA, USA) was equipped with Chemstation software (version number: B.04.03, Agilent Technologies), and LC5090 series (Zhejiang Fuli analytical instrument Co., Ltd., Wenling, China) was equipped with a Chemstation software (version number: 1.5.0.0, Zhejiang Fuli analytical instrument Co., Ltd.). The separations were performed on an Agilent Zorbax Extend reversed-phase C18 column (5 μm, 250 × 4.6 mm) purchased from Agilent (Santa Clara, CA, USA). The flow rate, the injection volume, and the column oven temperature were 1.0 mL/min, 2 mL, and 25°C, respectively. The detection wavelength was 281 nm. The mobile phase consisted of 0.1% (*v*/*v*) formic acid aqueous solution (A) and acetonitrile (B) with the gradient program as follows: 0–10 min, 7–17% of B; 10–16 min, 17–21% of B; 16–31 min, 21–21% of B; 32–40 min, 21–29% of B; 40–44 min, 29–35% of B; 44–45min, 35–100% of B. The dry matter contents were determined gravimetrically using hot air drying at 105 °C to a constant weight [26].

### 3.6. Data Analysis

The purity of phenolic compounds (*PC_Purity_*), including DSS, PA, CA, RA, LA, SAB, and SAA, were calculated using Equations (9) and (10):

(9) $$PC_{Purity}^{E}=C_{i}^{E}M_{i}^{E}/{DM}^{E}\times 100\%\;\left(i=1,\;2\;.\;.\;.\;,\;7\right)$$

(10) $$PC_{Purity}^{S}=C_{i}^{S}M_{i}^{S}/{DM}^{S}\times 100\%\;\left(i=1,\;2\;.\;.\;.\;,\;7\right)$$

where *C* and *M* refer to the concentration and mass, respectively; *DM* is the mass of dry matter; superscript *E* and *S* are the water extract and crude SAB solution, respectively; and subscript *i* refers to DSS, PA, CA, RA, LA, SAB, and SAA, respectively.

The yield of phenolic compounds (*PC_yield_*) were defined as in Equations (11) and (12):

(11) $$PC_{yield}^{E}=\frac{C_{i}^{E}M_{i}^{E}}{M_{DS}}$$

(12) $$PC_{yield}^{S}=\frac{C_{i}^{S}M_{i}^{S}}{M_{DS}}$$

where *M_DS_* is the mass of the Danshen.

SAB yield in crude SAB solution (${SAB}_{yield}^{S}$) and SAB purity in crude SAB solution (${SAB}_{purity}^{S}$) was considered as the process indices for the preparation processes of crude SAB. To reflect the comprehensive effects of process parameters on all evaluation indicators, the key parameters of crude SAB preparation process were identified by employing the method of the standard partial regression coefficient [27,28,29]. The response variables of *Y*_1_ and *Y*_2_ were standardized firstly according to following Equation (13). After that, the standard partial regression coefficients were calculated by multivariate linear regression based on the Equation (14). Finally, the absolute values of each standard partial regression coefficient were weighted and summed up to get *IF* values according to Equation (15). Parameters with higher *IF* values were expected to have greater influences on responses.

(13) $$Y_{k}^{\prime }=\frac{Y_{k}-{\bar{Y}}_{k}}{STD_{k}}\left(k=1,2\right)$$

(14) $$Y_{k}^{\prime }=\beta_{0,k}+\overset{7}{\underset{j=1}{\sum }}\beta_{j,k}X_{j}\left(j=1,\;2,\ldots ,\;7\right)$$

(15) $$IF_{j}=\overset{2}{\underset{k=1}{\sum }}w_{k}\left|\beta_{j,k}\right|$$

where *Y_k_* is the measured value; *Y’_k_* and ${\bar{Y}}_{k}$ are the standardized value and average value of each response, respectively; *STD_k_* is the standard deviation of each response; and number *k* (*k* = 1, 2) represents ${SAB}_{yield}^{S}$ and ${SAB}_{purity}^{S}$ in crude SAB solution. β*_0,k_* is a constant term, *X_j_* is the coded value of a parameter, subscript *j* refers to a process parameter, and *β_j,k_* is the standard partial regression coefficient. In this study, each response was considered equally important, which signifies that the *w_k_* values were both 1/2.

The data of Box–Behnken-designed experiments were modeled with Equation (16) in terms of the coded factors below:

(16) $$Y=a_{0}+\overset{3}{\underset{i=1}{\sum }}a_{i}X_{i}+\overset{3}{\underset{i=1}{\sum }}a_{ii}X_{i}^{2}+\overset{3}{\underset{j=i+1}{\sum }}a_{ij}X_{i}X_{j}\left(i=1,\;2,\;3;\;j=2,\;3\right)$$

where *a_0_* is a constant; *a_i_*, *a_ii_*, and *a_ij_* are the regression coefficients for the linear, quadratic, and interaction of the model, respectively. The meaning of variable *X* in Equation (14) and Equation (16) was the same. Stepwise regression was carried out to establish quantitative models between process indices and key parameters of crude SAB preparation process. The significance level for moving in or out a term was set to 0.1. The Design Expert 8.0.6 software (Stat-Ease Inc., Minneapolis, MN, USA) was used for the calculations.

## 4. Conclusions

In this work, SAB disodium salt was successfully prepared from Danshen through a series of processes including water extraction, concentration, acidification, 1-butanol extraction, water washing, basification, water back extraction, chromatography, and drying. Three parameters of extraction temperature, extraction pH, and back-extraction pH were found to be significantly affect the SAB purity and yield of the crude SAB solution. The optimized ranges of these parameters were given. CG161M resin was selected for further purification of crude SAB solution due to its high adsorption capacity, high desorption ratio, and fast adsorption kinetics. The conditions of chromatography were also optimized. Water was used as the eluent for the purification of SAB disodium salt. It is low cost and environment-friendly. About 5 g of high-purity SAB disodium salt (>95%) could be obtained in one preparation process. The productivity of the present method was larger compared with other reported works. 

Different batches of Danshen were used to prepare SAB disodium salt with the optimized parameters. Water extract indices were considered to represent the effects of Danshen quality and water extraction process. Therefore, phenolic compound purity and phenolic compound yield of water extracts were measured. After the quantitative relationships between SAB disodium salt purity and yield and water extract indices were established, the quality standard of Danshen water extract was calculated and verified. With the setting of intermediate quality standards, product quality can be predicted at an early stage of manufacturing processes. The method used in this work can also be applied to develop process intermediate quality standards for other natural products.

## Figures and Tables

**Table 1:** Plackett–Burman design conditions and results.

Run	Process Parameters							Process Indices	
	*X*_1_ (°C)	*X*_2_	*X*_3_	*X*_4_ (h)	*X*_5_ (mL/g)	*X*_6_ (mL)	*X*_7_ (mL)	*Y*_1_ (mg/g Medicinal Slices)	*Y*_2_ (%)
1	50	4.2	5.0	2.0	8.0	60	100	0.516	37.7
2	50	2.4	4.0	2.0	10.0	80	100	2.72	53.9
3	100	2.4	5.0	4.0	10.0	60	50	9.23	38.7
4	75	3.3	4.5	3.0	9.0	70	75	5.17	65.2
5	50	2.4	4.0	2.0	8.0	60	50	2.69	57.2
6	50	4.2	5.0	4.0	8.0	80	50	0.970	58.1
7	100	2.4	5.0	2.0	8.0	80	50	10.2	49.0
8	100	2.4	4.0	4.0	8.0	60	100	5.09	45.9
9	75	3.3	4.5	3.0	9.0	70	75	5.33	63.8
10	100	4.2	5.0	2.0	10.0	60	100	0.881	33.1
11	50	2.4	5.0	4.0	10.0	80	100	10.8	64.2
12	75	3.3	4.5	3.0	9.0	70	75	5.61	62.9
13	100	4.2	4.0	2.0	10.0	80	50	0.300	38.8
14	100	4.2	4.0	4.0	8.0	80	100	0.238	28.5
15	50	4.2	4.0	4.0	10.0	60	50	0.205	39.5

X_1_: extraction temperature; X_2_: extraction pH; X_3_: back-extraction pH; X_4_: extraction time; X_5_: water consumption for extraction; X_6_: 1-butanol amount; X_7_: water consumption for washing; Y_1_: SAB yield in crude SAB solution; Y_2_: SAB purity in crude SAB solution.

**Table 2:** Box–Behnken design conditions and results.

Run	Key Parameters of Crude SAB Preparation Process			Process Indices	
	*X*_1_ (°C)	*X*_2_	*X*_3_	*Y*_1_ (mg/g Medicinal Slices)	*Y*_2_ (%)
S1	100	3.3	4.0	1.59	47.0
S2	75	3.3	4.5	5.46	59.2
S3	75	2.4	5.0	10.5	54.1
S4	100	3.3	5.0	7.67	48.9
S5	50	2.4	4.5	5.28	53.6
S6	100	2.4	4.5	8.19	43.5
S7	100	4.2	4.5	0.741	50.2
S8	50	3.3	4.0	1.06	55.7
S9	50	4.2	4.5	0.506	52.1
S10	75	3.3	4.5	5.26	59.4
S11	75	4.2	4.0	0.227	46.8
S12	50	3.3	5.0	5.11	57.8
S13	75	2.4	4.0	2.72	43.9
S14	75	3.3	4.5	5.55	59.9
S15	75	4.2	5.0	1.09	44.2
S16	75	3.3	4.5	6.07	58.1

**Table 3:** Pseudo-second-order kinetics parameters of resins calculated on the basis of SAB.

Resin	Pseudo-Second-Order		
	*Q_e_*(cal) (mg/g Dry Resin)	*k_2_* (min^−1^)	R^2^
CG161M	12.55	0.103	0.99
50 W × 2	7.519	0.00900	0.96
50 W × 4	11.74	0.00479	0.95

*Q_e_*: the adsorption capacity at equilibrium; *k_2_*: the rate constant of pseudo-second-order model.

**Table 4:** The effects of yield and purity of phenolic compounds on ${\mathrm{SAB}}_{\mathrm{purity}}^{F}$ and ${\mathrm{SAB}}_{\mathrm{yield}}^{F}.$

Run	Habitat	Phenolic Compounds Yield of Water Extract							Phenolic Compounds Purity of Water Extract							Final Product Indices	
		$DSS_{yield}^{E}\;(\mathrm{mg}/g\;\mathrm{medicinal}\;\mathrm{slices})$	$PA_{yield}^{E}\;(\times 10^{-2}\mathrm{mg}/g\;\mathrm{medicinal}\;\mathrm{slices})$	$CA_{yield}^{E}\;(\times 10^{-2}\mathrm{mg}/g\;\mathrm{medicinal}\;\mathrm{slices})$	$RA_{yield}^{E}\;(\mathrm{mg}/g\;\mathrm{medicinal}\;\mathrm{slices})$	$LA_{yield}^{E}\;(\mathrm{mg}/g\;\mathrm{medicinal}\;\mathrm{slices})$	$SAB_{yield}^{E}\;(\mathrm{mg}/g\;\mathrm{medicinal}\;\mathrm{slices})$	$SAA_{yield}^{E}\;(\mathrm{mg}/g\;\mathrm{medicinal}\;\mathrm{slices})$	$DSS_{purity}^{E}\;(\times 10^{-2},\;\%)$	$PA_{purity}^{E}\;(\times 10^{-2},\;\%)$	$CA_{purity}^{E}(\times 10^{-2},\;\%)$	$RA_{purity}^{E}\;(\%)$	$LA_{purity}^{E}\;(\%)$	$SAB_{purity}^{E}\;(\%)$	$SAA_{purity}^{E}\;(\%)$	$SAB_{purity}^{F}\;(\%)$	$SAB_{yield}^{F}\;(\mathrm{mg}/g\;\mathrm{medicinal}\;\mathrm{slices})$
Ⅰ	Bozhou, Anhui Province	0.295	1.08	5.54	1.15	0.685	22.6	0.301	9.81	0.358	1.84	0.381	0.228	7.53	0.100	95.9	1.42
Ⅱ	Minxian, Gansu Province	0.428	2.54	27.9	1.34	0.0746	0.809	0.0322	23.7	1.41	15.4	0.742	0.0413	0.447	0.0178	22.5	0.00483
Ⅲ	Wanrong, Shanxi Province	0.252	2.61	8.30	0.428	0.707	21.1	0.392	7.00	0.725	2.31	0.119	0.196	5.87	0.109	97.5	0.717
Ⅳ	Dancheng, Henan Province	0.377	1.92	6.40	0.709	0.976	20.1	0.372	12.1	0.617	2.05	0.228	0.313	6.45	0.119	88.3	0.784
Ⅴ	Zhongjiang, Sichuan Province	0.310	1.83	7.35	0.452	0.845	11.2	0.230	15.7	0.928	3.72	0.229	0.428	5.66	0.116	99.2	0.659
Ⅵ	Wanrong, Shanxi Province (1)	0.359	0.923	6.43	0.238	0.800	11.3	0.132	10.7	0.275	1.92	0.0710	0.238	3.38	0.0393	96.0	0.559
Ⅶ	Wanrong, Shanxi Province (2)	0.322	0.842	6.20	0.231	0.781	11.1	0.128	9.83	0.257	1.89	0.0704	0.238	3.38	0.0391	96.7	0.492
Ⅷ	Wanrong, Shanxi Province (3)	0.333	0.904	6.50	0.249	0.821	11.6	0.136	10.0	0.271	1.95	0.0746	0.246	3.50	0.0409	98.1	0.564

DSS: Danshensu; PA: protocatechuic aldehyde; CA: caffeic acid; RA: rosmarinic acid; LA: lithospermic acid; SAA: salvianolic acid A.

**Table 5:** The effects of ${\mathrm{SAB}}_{\mathrm{yield}}^{E}$ on ${\mathrm{SAB}}_{\mathrm{yield}}^{F}$ and ${\mathrm{CA}}_{\mathrm{purity}}^{E}$ on ${\mathrm{SAB}}_{\mathrm{purity}}^{F}.$

Habitat	$SAB_{yield}^{E}\;(\mathrm{mg}/g\;\mathrm{Medicinal}\;\mathrm{Slices})$	$CA_{purity}^{E}\;(\times 10^{-2},\;\%)$	$SAB_{purity}^{F}/\mathrm{PV}\;(\%)$	$SAB_{yield}^{F}/\mathrm{PV}\;(\mathrm{mg}/g\;\mathrm{Medicinal}\;\mathrm{Slices})$
Julu, Hebei Province	10.30	2.31	99.3/95.3	0.732/0.487

PV predicted values.

## Supplementary Materials

Supplementary materials are available online at https://www.mdpi.com/1420-3049/24/7/1269/s1. Table S1: Partial regression coefficients, importance factors, and coefficient confidence intervals, Table S2: Estimated parameter values, determination coefficients, ANOVA results, and coefficient confidence intervals of quadratic models, Table S3: Physical properties of the test macroporous resins; Figure S1: FTIR spectrum of SAB disodium salt and SAB, Figure S2: TOF-MS spectrum of SAB disodium salt.
