# Supplementary material for: Preparation of Salvianolic Acid B Disodium Salt Considering the Water Extract Quality Standard
Source: Molecules. 2019 Apr 1;24(7):1269. doi: 10.3390/molecules24071269 (PMC6479940; doi:10.3390/molecules24071269)
Supplement: Supplementary file 1 [file molecules-24-01269-s001.pdf]

Table S1. Partial regression coefficients, importance factors, and coefficient confidence intervals

Factor	Y_1			Y_2			IF_j Value
	Coefficient	95% CI Low	95% CI High	Coefficient	95% CI Low	95% CI High	
X_1	0.180	-0.140	0.500	-0.510	-1.180	0.160	0.345
X_2	-0.840	-1.150	-0.520	-0.490	-1.160	0.180	0.665
X_3	0.480	0.160	0.790	0.110	-0.560	0.790	0.295
X_4	0.210	-0.110	0.520	0.034	-0.640	0.710	0.122
X_5	0.099	-0.220	0.410	-0.055	-0.730	0.620	0.077
X_6	0.150	-0.170	0.460	0.270	-0.400	0.940	0.210
X_7	-0.075	-0.390	0.240	-0.120	-0.790	0.550	0.098

X_1: extraction temperature; X_2: extraction pH; X_3: back extraction pH; X_4: extraction time; X_5: water consumption for extraction; X_6: 1-butanol amount; X_7: water consumption for washing; Y_1: SAB yield in crude SAB solution; Y_2: SAB purity in crude SAB solution; IF: importance factor; j: a process parameter. CI: confidence interval.

Table S2. Estimated parameter values, determination coefficients, ANOVA results, and coefficient confidence intervals of quadratic models

Term	Y_1				Y_2			
	Coefficient	95% CI Low	95% CI High	p value	Coefficient	95% CI Low	95% CI High	p value
Constant	5.59	5.19	5.99	-	58.11	55.56	60.65	-
X_1	0.78	0.50	1.06	0.0005	-3.71	-5.79	-1.63	0.0024
X_2	-3.02	-3.30	-2.74	< 0.0001	-	-	-	-
X_3	2.35	2.07	2.63	< 0.0001	-	-	-	-
X_1X_2	-0.67	-1.07	-0.27	0.0063	-	-	-	-
X_1X_3	0.51	0.11	0.91	0.0209	-	-	-	-
X_2X_3	-1.73	-2.13	-1.34	< 0.0001	-3.19	-6.13	-0.25	0.0357
X_1^2	-0.84	-1.24	-0.44	0.0021	-	-	-	-
X_2^2	-1.06	-1.46	-0.67	0.0006	-7.22	-10.16	-4.28	0.0002
X_3^2	-0.88	-1.28	-0.49	0.0016	-4.69	-7.63	-1.75	0.0049
R^2	0.9957				0.8509			
p value	< 0.0001				0.0002			

Table S3. Physical properties of the test macroporous resins

Trade name	Type	Particle diameter (mm)	Moisture content (%)
Polyamide (30-60 mesh)	/	0.595-0.250	51.36
HPD100 [1]	Non-polar	0.30–1.25	66.19
AB-8 [1]	Weak-polar	0.30–1.25	62.44
FPA 98Cl	Strong base anion	0.63-0.85	69.27
FPA 90Cl	Strong base anion	0.65-0.82	61.24
FPA 53	Weak base anion	0.50-0.75	60.37
50W×2 (200-400 mesh)	Strong acid cation	0.035-0.075	67.08
50W×4 (200-400 mesh)	Strong acid cation	0.035-0.075	67.20
CG161M	Non-polar	0.075	70.44
XAD 1600N	Non-polar	0.35-0.45	71.04

1. Jia, G.T.; Lv, X.Y.; Enrichment and purification of madecassoside and asiaticoside from *Centella asiatica* extracts with macroporous resins. *J. Chromatogr. A* 2008, *1193*, 136-141.

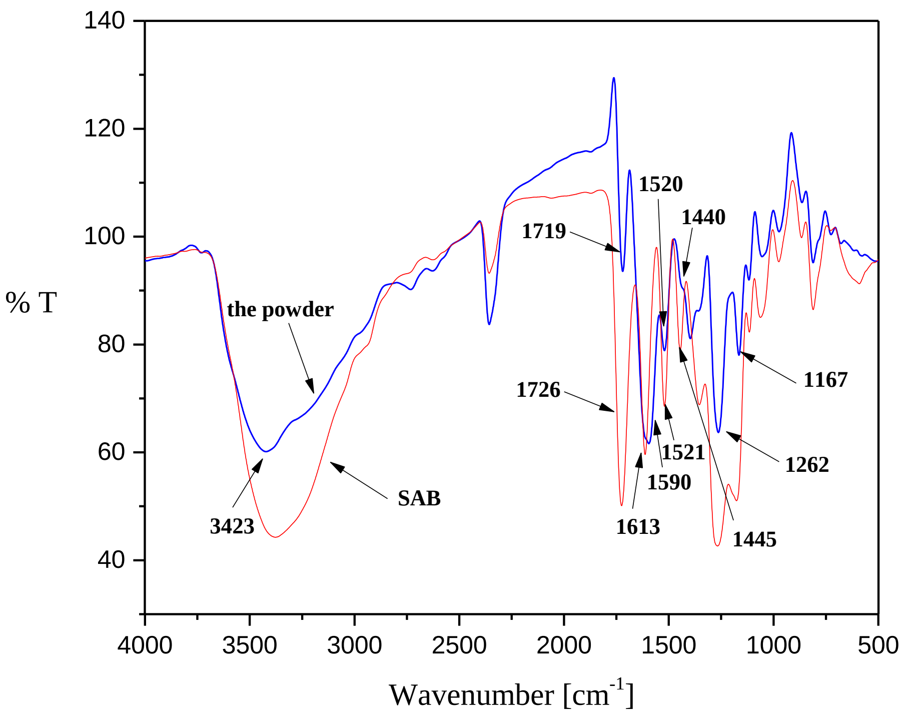

Figure S1. FTIR spectrum of the SAB disodium salt and SAB

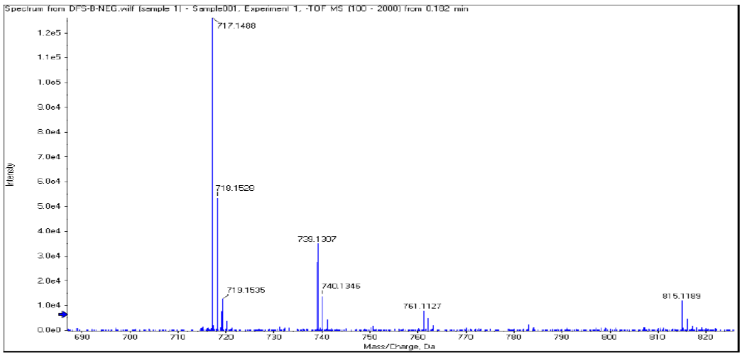

Figure S2. TOF-MS spectrum of the SAB disodium salt
